# Gut Microbiomes of Two Bamboo Locusts Reveal Divergent Metabolic Potentials

**DOI:** 10.1002/ece3.74143

**Published:** 2026-08-28

**Authors:** Hong‐Yu Liao, Ye‐Song Ren, Xi Cheng, Hai‐Tao Huang, Meng‐Zhi Jin, Yang Zeng, Dao‐Hong Zhu

**Affiliations:** ^1^ Laboratory of Insect Behavior and Evolutionary Ecology, College of Life Sciences Central South University of Forestry and Technology Changsha Hunan China; ^2^ College of Agriculture and Biotechnology Hunan University of Humanities, Science and Technology Loudi Hunan China; ^3^ Key Laboratory of Development, Utilization Quality and Safety Control of Characteristic Agricultural Resources in Central Hunan Province Loudi Hunan China; ^4^ Loudi Institute of Agricultural Sciences Loudi Hunan China

**Keywords:** 16S rRNA, *Ceracris kiangsu*, *Ceracris nigricornis*, functional prediction, gut microbiota, microbial diversity

## Abstract

The yellow‐spined bamboo locust (*Ceracris kiangsu*) and the green‐spined bamboo locust (*Ceracris nigricornis*) are two common bamboo‐feeding forestry pests in China. *C. kiangsu* has stronger migratory capacity and causes greater damage, yet comparative studies of their gut microbiota are lacking. Here, we profiled the gut microbial communities of female *C. kiangsu* from six geographic populations using 16S rRNA gene amplicon sequencing (Illumina MiSeq) and compared these data with publicly available gut microbiome datasets of female *C. nigricornis* from NCBI. At the phylum level, the gut microbiota of both species was dominated by Firmicutes and Proteobacteria. At the genus level, dominant taxa included *Klebsiella* and *Achromobacter*. Despite these similarities, the two species differed significantly in microbial diversity. Alpha‐diversity indices showed highly significant differences in richness and diversity (*p* < 0.01), and beta‐diversity analyses (PCoA) revealed clear separation of community structure between species (PERMANOVA, *p* < 0.001). Functional prediction using PICRUSt2 indicated that, at KEGG Level 2, pathways related to carbohydrate and lipid metabolism were significantly enriched in *C. kiangsu* compared with *C. nigricornis* . At KEGG Level 3, *C. kiangsu* also showed higher predicted abundances of starch and sucrose metabolism, glycolysis/gluconeogenesis, the pentose phosphate pathway, and pyruvate metabolism. Collectively, these results reveal distinct gut microbial communities and predicted functional profiles between the two species. The enrichment of predicted carbohydrate‐ and lipid‐related pathways in *C. kiangsu* generates a testable hypothesis that its gut microbiota may be associated with host energy metabolism, but direct links to flight performance or ecological fitness require further experimental validation.

## Graphical abstract

We compared the gut microbiota of two bamboo‐feeding locust pests, *Ceracris kiangsu* and *Ceracris nigricornis*, using 16S rRNA amplicon sequencing for six geographic populations of *C. kiangsu* and publicly available *C. nigricornis* datasets. Both species were dominated by Firmicutes and Proteobacteria, but showed significant differences in community diversity and structure. PICRUSt2 functional prediction indicated enrichment of carbohydrate‐ and lipid‐metabolism pathways in *C. kiangsu*, suggesting greater metabolic potential that may contribute to its stronger flight capacity and ecological fitness.

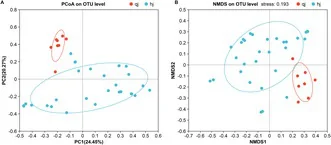

## 1 Introduction

Insects are widely distributed and highly diverse. Their gut—typically divided into the foregut, midgut, and hindgut—supports digestion, nutrient uptake, and waste excretion (Schmidt and Engel 2021). The insect gut also hosts large microbial communities that form close associations with the host and contribute to key processes such as nutrient acquisition and immune defense (Linser and Dinglasan 2014). With the rapid development of high‐throughput sequencing, insect gut microbiomes have become an active research area. Within Orthoptera, studies on gut microbiota have increased in recent years, but research on the bamboo locust genus *Ceracris* remains limited (Huang et al. 2021).

The yellow‐spined bamboo locust (*Ceracris kiangsu*) and the green‐spined bamboo locust (*Ceracris nigricornis*) are among the most destructive *Ceracris* pests in China (Fan et al. 2014). They are widely distributed across regions where major bamboo hosts (e.g., *Phyllostachys* spp. and *Bambusa* spp.) occur, including Yunnan, Sichuan, Guangxi, Guangdong, Hunan, and Hubei (Yu and Shen 2011). Compared with related species, *C. kiangsu* appears to have broader ecological tolerance and higher fitness, enabling long‐distance dispersal and access to new resources. When it enters new habitats, it often competes strongly with closely related species occupying similar niches, such as *C. nigricornis* , and frequently becomes the dominant species (Liu et al. 2021). Notably, *C. kiangsu* has been reported as the first orthopteran insect showing “mud‐puddling” behavior, which may help replenish minerals (Liu et al. 2021). Together with its strong environmental adaptability, this species has become a major threat to bamboo forests and has been referred to as the “second most destructive forest pest in China” after pine caterpillars. However, evidence from the gut microbiome perspective is still lacking to help explain why *C. kiangsu* tends to outperform *C. nigricornis* .

The composition of gut microbial communities in herbivorous insects can be shaped by host traits, diet, environmental conditions, and microbial transmission processes (Engel and Moran 2013), while closely related species may compete intensely because they share similar resources and niches (Li et al. 2025). Beyond differences in host genomes, symbiotic gut microbes may also influence host fitness and ecological performance (Hardin 1960). Previous studies have shown that gut microbiota vary across insect species (Malacrinò 2022) and can also differ across developmental stages (Costa‐Martínez et al. 2026). For example, the gut microbiota of *Bactrocera dorsalis* across larval, pupal, and adult stages is dominated by Proteobacteria, Firmicutes, and Bacteroidetes (Zhu et al. 2022). Larvae of the diamondback moth show high proportions of Proteobacteria and Firmicutes, among other groups (Douglas 2015). In black soldier fly larvae, Actinobacteria, Bacteroidetes, Firmicutes, and Proteobacteria are commonly abundant (Ren et al. 2022). Similar core groups are reported across stages of *Zeugodacus tau* (Azis et al. 2019) and in the planthopper *Laodelphax striatellus* (Xia et al. 2018). In locusts, dominant gut bacteria also frequently belong to Proteobacteria and Firmicutes (Wang et al. 2022). These observations suggest that Firmicutes and Proteobacteria are widespread and often dominant in insect guts. Importantly, Firmicutes may provide functional benefits: they can contribute to nitrogen recycling in *B. dorsalis* (Ren et al. 2022), increase resistance to organophosphate insecticides and improve fitness in the diamondback moth, and support the breakdown of plant cell wall components such as cellulose and lignin.

For *Ceracris*, available evidence is still scarce. One study by Huang et al. (2021) examined the relationship between bamboo‐feeding insects and host plants and reported that host evolutionary history and diet can shape gut microbial communities, including in *C. nigricornis* . Differences among bamboo locust species suggest that host genetic background may be a key factor influencing gut microbial structure, consistent with broader patterns reported across insects (Malacrinò 2022; Zhu et al. 2022). Given the clear ecological differences between *C. kiangsu* and *C. nigricornis* , we hypothesized that their gut microbiota also differ in composition and predicted function, and that these differences may contribute to the stronger dispersal ability and ecological performance of *C. kiangsu*. In this study, we used high‐throughput sequencing to compare gut microbial diversity, taxonomic composition, and predicted functional profiles between *C. kiangsu* and *C. nigricornis* , providing baseline data for understanding how gut microbiota may relate to host adaptation and ecological success.

## 2 Materials and Methods

### 2.1 Sample Collection

Adult female yellow‐spined bamboo locusts (*Ceracris kiangsu*) were collected from six locations in China: Mengla, Xishuangbanna, Yunnan Province (22.03° N, 101.5° E); Guangning, Zhaoqing, Guangdong Province (23.73° N, 112.5° E); Xing'an, Guilin, Guangxi Autonomous Region (25.64° N, 110.49° E); Suxian District, Chenzhou, Hunan Province (25.75° N, 113.24° E); Taojiang, Yiyang, Hunan Province (28.25° N, 112.01° E); and Chibi, Xianning, Hubei Province (29.55° N, 113.71° E). Immediately after collection, individuals were frozen on dry ice. For each population, five replicate samples were prepared, and each sample consisted of three adult females. After the samples arrived at the laboratory, the insects were dissected and the tissues were stored at −80°C until use.

For comparative analysis, raw 16S rRNA gene amplicon sequencing reads from adult female *Ceracris nigricornis* were downloaded from the NCBI database under accession number DRA009676. The original *C. nigricornis* specimens were collected in Lin'an, Zhejiang Province, China (30.33° N, 119.47° E). Previously processed OTU tables, taxonomic profiles, diversity indices, and functional prediction results from the original publication were not used. Instead, the raw FASTQ files of *C. nigricornis* were processed de novo together with the newly generated raw sequencing reads of *C. kiangsu* using the same bioinformatic workflow. Bioinformatic processing was performed by Shanghai Majorbio Bio‐Pharm Technology Co. Ltd. (Shanghai, China).

### 2.2 DNA Extraction and Sequencing

Gut samples from *Ceracris kiangsu* were processed under sterile conditions using disinfected dissection tools. Each gut sample was homogenized in a sterile grinder, and total DNA was extracted following the manufacturer's protocol. The V3–V4 region of the bacterial 16S rRNA gene was amplified using barcoded universal primers 338F (5′‐ACTCCTACGGGAGGCAGCAG‐3′) and 806R (5′‐GGACTACHVGGGTWTCTAAT‐3′) (Huang et al. 2021).

PCR reactions were performed in 20 μL volumes containing 4 μL 5× FastPfu buffer, 2 μL 2.5 mM dNTPs, 0.8 μL each primer (5 μM), 0.4 μL FastPfu DNA polymerase, ~10 ng template DNA, and nuclease‐free water to volume. Cycling conditions were: 95°C for 3 min; 27 cycles of 95°C for 30 s, 55°C for 30 s, and 72°C for 30 s; followed by 72°C for 10 min and a final hold at 4°C. Each sample was amplified in triplicate, and the three reactions were pooled to reduce variation. Amplicons were checked on a 2% agarose gel, gel‐purified using the AxyPrep DNA Gel Extraction Kit (Axygen), and quantified with a Quantus Fluorometer (Promega).

### 2.3 Library Preparation and Sequencing

Under Purified amplicons were used for library construction. Libraries were prepared with the NEXTFLEX Rapid DNA‐Seq kit following the manufacturer's workflow: adapter ligation, bead‐based cleanup to remove unligated products, PCR enrichment, and final bead purification. Qualified libraries were sequenced on an Illumina MiSeq platform (paired‐end 300 bp; MiSeq PE300) by Shanghai Majorbio Bio‐Pharm Technology Co. Ltd. Raw sequence data have been deposited in the China National GeneBank DataBase (CNGBdb) under accession number CNP0008191.

### 2.4 Functional Prediction

Raw sequencing reads from both *C. kiangsu* and *C. nigricornis* were processed de novo using the same bioinformatic workflow, including quality filtering, paired‐end read merging, OTU clustering, taxonomic assignment, diversity analysis, and functional prediction. Raw reads were quality‐filtered using fastp version 0.19.6. Briefly, low‐quality bases at read ends were trimmed using a sliding window of 50 bp (reads were truncated when the average quality score fell below Q20). Reads shorter than 50 bp after trimming and reads containing ambiguous bases (N) were removed. Paired‐end reads were merged using FLASH with a minimum overlap of 10 bp and a maximum mismatch rate of 0.2. Reads were assigned to samples based on barcode and primer sequences (barcode mismatches not allowed; up to two primer mismatches permitted), and sequence orientation was adjusted accordingly.

Following quality control, valid sequences from all samples of both species were subjected to unified clustering into operational taxonomic units (OTUs) using UPARSE version 7.1 (Edgar 2013) at 97% sequence similarity, and chimeric sequences were removed during clustering. A common OTU abundance table was subsequently generated for comparative analyses. This unified OTU‐based framework provided a common feature space for consistent community‐level comparisons between the two datasets. The resulting OTUs were treated as operational sequence clusters rather than definitive bacterial species. OTUs annotated as host mitochondria or plant chloroplasts were excluded. To minimize the impact of uneven sequencing depth, all samples were rarefied to the same number of reads (the minimum sample size). After rarefaction, Good's coverage exceeded 99% for all samples, indicating sufficient sequencing depth. Representative OTU sequences were taxonomically assigned using the RDP Classifier version 2.11 (Wang et al. 2007) against the Silva 16S rRNA database (v138) with a confidence threshold of 70%. The same threshold was applied uniformly to both datasets, and genus‐ and species‐level assignments were interpreted cautiously. Predicted functional profiles were inferred from the 16S data using PICRUSt2 version 2.2.0 (Douglas et al. 2020) based on the OTU abundance table.

### 2.5 Statistical Analysis

Bioinformatic and statistical analyses were implemented through the Majorbio Cloud Platform, which served as the computational interface. To improve transparency and reproducibility, the underlying software, database versions, analytical parameters, distance metrics, and statistical thresholds used in each analysis are specified below. Alpha diversity (Observed OTUs, Chao1, Shannon, Simpson, ACE, and Good's coverage) was calculated using mothur (http://www.mothur.org/wiki/Calculators) (Schloss et al. 2009). Differences in alpha diversity between *C. kiangsu* and *C. nigricornis* were evaluated using the Wilcoxon rank‐sum test.

Beta diversity was assessed using Bray–Curtis distances and visualized by principal coordinates analysis (PCoA) and non‐metric multidimensional scaling (NMDS). Group differences in community structure were tested using PERMANOVA (permutational multivariate analysis of variance). Differentially abundant taxa across taxonomic levels were identified using LEfSe (linear discriminant analysis effect size) (http://huttenhower.sph.harvard.edu/LEfSe) (Segata et al. 2011) with an LDA threshold > 4 and *p* < 0.05. In addition, distance‐based redundancy analysis (db‐RDA) based on Bray–Curtis distances was used to evaluate the extent to which host species explained variation in gut community structure.

## 3 Results and Discussion

### 3.1 Landscape of Gut Microbiome Diversity

Illumina MiSeq sequencing generated 1,368,262 high‐quality reads from 30 gut samples of *Ceracris kiangsu*, with an average of 45,609 reads per sample. After quality filtering, the total number of bases was approximately 5.86 × 10^8^ and the mean read length was 428 bp. For *C. nigricornis* , 1,069,683 high‐quality reads were obtained with a mean read length of 442 bp. At a 97% sequence similarity threshold, 1605 OTUs were initially identified across all samples. After removing chimeras and sequences assigned to host mitochondria and plant chloroplasts, 1518 bacterial OTUs remained for downstream analyses.

The two species differed markedly in OTU richness. *C. kiangsu* contained 362 OTUs, whereas *C. nigricornis* contained 1290 OTUs. Only 134 OTUs were shared between species; 228 OTUs were unique to *C. kiangsu* and 1156 were unique to *C. nigricornis* , indicating substantially higher bacterial richness in *C. nigricornis* . Taxonomic assignment placed these OTUs into 28 phyla, 64 classes, 151 orders, 239 families, and 388 genera. In *C. kiangsu*, bacteria were distributed across 25 phyla, 51 classes, 104 orders, 163 families, and 257 genera. In contrast, *C. nigricornis* included 20 phyla, 47 classes, 113 orders, 177 families, and 268 genera (Table S1). Together, these results suggest that *C. nigricornis* shows higher OTU richness, while its taxa are concentrated within fewer higher‐level lineages, consistent with diversification within a limited number of dominant phyla.

**FIGURE 1:**
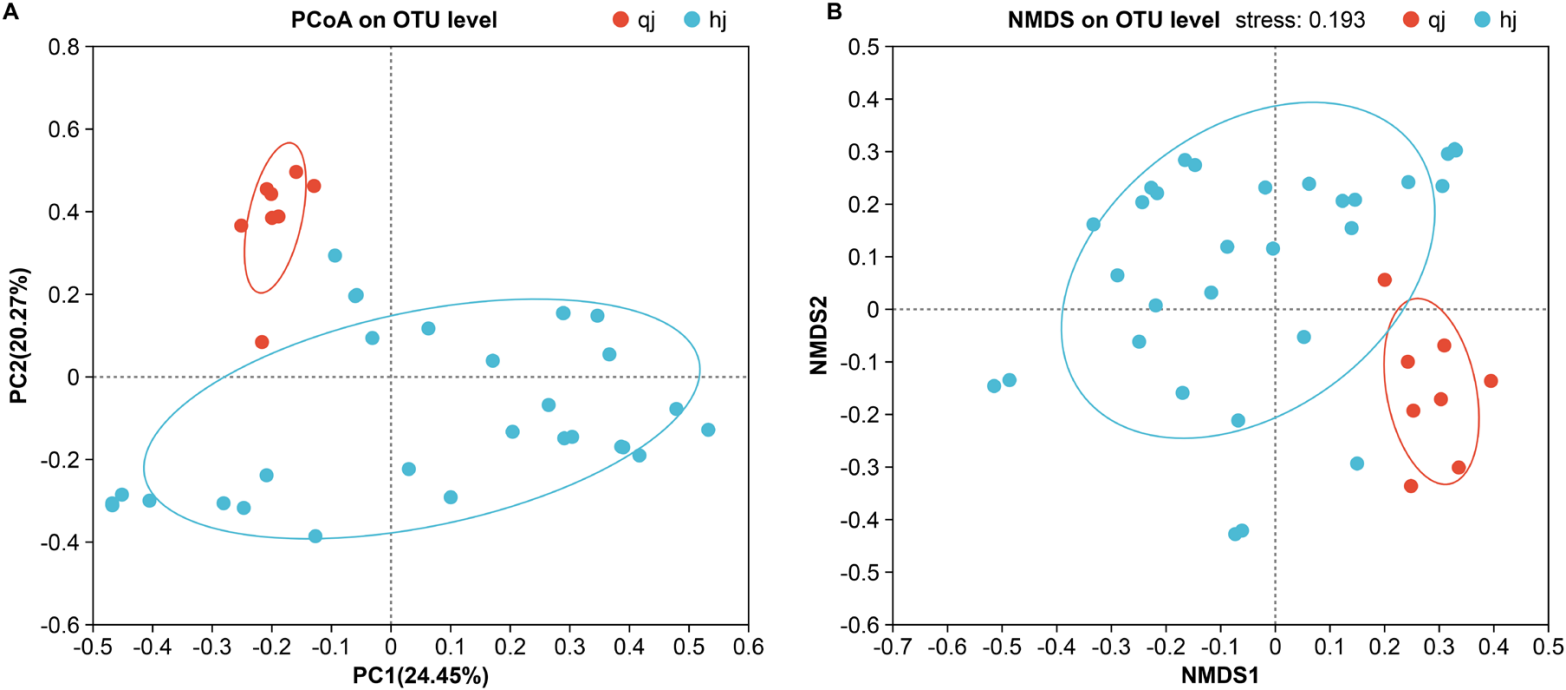
Beta‐diversity analysis of gut bacterial communities in *C. kiangsu* (hj) and *C. nigricornis* (qj). Based on Bray–Curtis dissimilarities of bacterial abundances, (A) principal coordinates analysis (PCoA) and (B) non‐metric multidimensional scaling (NMDS) are shown.

Alpha‐diversity analyses further supported significant differences between species. Richness metrics (Sobs, Chao1, ACE) and diversity indices (Shannon, Simpson) differed strongly between *C. kiangsu* and *C. nigricornis* (Wilcoxon test, *p* < 0.01; Table S2), indicating differences in both richness and evenness. Beta‐diversity analyses based on Bray–Curtis dissimilarity showed clear separation between species: PCoA clustered samples by species with minimal overlap (Figure 1A), and NMDS also revealed distinct community structures (stress = 0.05) with significant between‐group differences confirmed by PERMANOVA (*p* < 0.001) (Figure 1B). Overall, both alpha and beta diversity consistently demonstrate that the gut microbiomes of *C. kiangsu* and *C. nigricornis* are significantly different. This pattern agrees with previous findings that host species identity is a major driver of insect gut microbiome structure (Malacrinò 2022; Zhu et al. 2022).

### 3.2 Differences in Taxonomic Composition of the Gut Microbiota

**FIGURE 2:**
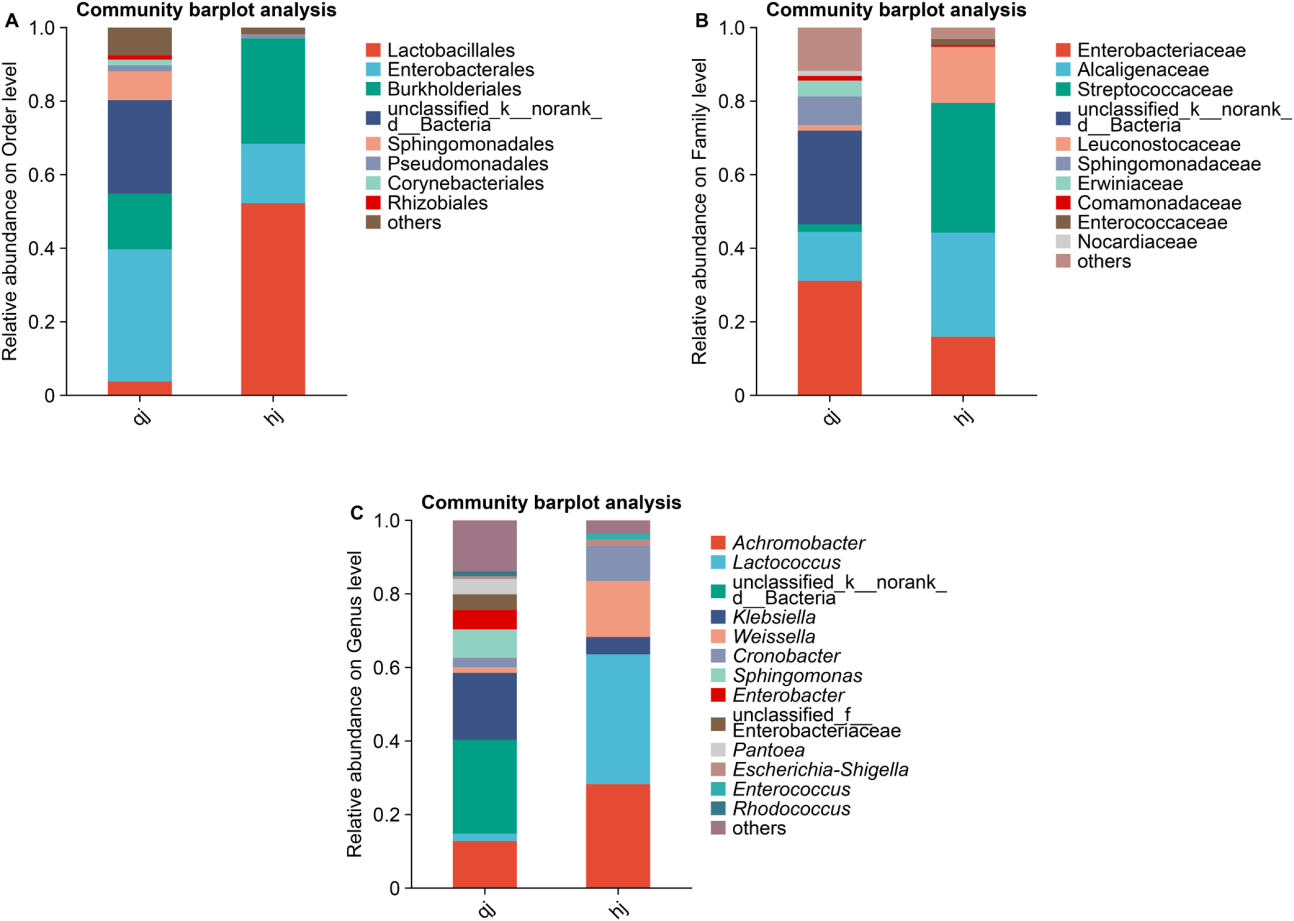
Relative abundances of gut microbial communities in the *C. kiangsu* (hj) and the *C. nigricornis* (qj) at the order (A), family (B), and genus (C) levels. Each color represents a taxon in each panel. The color labeled “Others” denotes taxa at the corresponding taxonomic rank that are not shown individually.

We next compared gut microbial composition and relative abundance between the two species. At the phylum level, both microbiomes were dominated by Firmicutes and Proteobacteria, but their proportions differed substantially (Figure 2A). In *C. kiangsu*, Firmicutes accounted for 53.45% and Proteobacteria for 46.35%, together comprising ~99.8% of the community. In *C. nigricornis* , Proteobacteria dominated (69.88%), Firmicutes was much lower (4.83%), and Actinobacteriota accounted for 2.90%; all remaining phyla were below 2% in relative abundance. Thus, *C. nigricornis* had a broader phylum‐level profile but was strongly Proteobacteria‐driven, whereas *C. kiangsu* was almost entirely composed of Firmicutes and Proteobacteria.

This dominance pattern is broadly consistent with reports from other Orthoptera, including migratory locusts and desert locusts, where Proteobacteria and Firmicutes often represent the core gut lineages (Wang et al. 2020; Lavy et al. 2019; Bai et al. 2022). Notably, the much higher proportion of Firmicutes in *C. kiangsu* may imply stronger enrichment of fermentative or carbohydrate‐processing bacteria, whereas the microbiome of *C. nigricornis* is more heavily weighted toward Gram‐negative taxa.

At finer taxonomic resolution, the two species differed in their dominant lineages (Figure S1). At the class level, *C. kiangsu* was dominated by Bacilli (53.41%) and Gammaproteobacteria (46.28%), while *C. nigricornis* was dominated by Gammaproteobacteria (59.00%) and Alphaproteobacteria (10.88%), with Bacilli at only 4.82%; Actinobacteria represented 2.46%. At the order level, the main groups in *C. kiangsu* were Lactobacillales (52.71%), Burkholderiales (28.97%), and Enterobacterales (16.13%). In *C. nigricornis* , Enterobacterales (39.78%) and Burkholderiales (16.49%) were most abundant, followed by Sphingomonadales (9.23%), while Lactobacillales was low (4.12%).

At the family level, *C. kiangsu* was dominated by Streptococcaceae (35.55%), Alcaligenaceae (28.53%), Enterobacteriaceae (16.01%), and Leuconostocaceae (15.46%). In *C. nigricornis* , Enterobacteriaceae (34.55%), Alcaligenaceae (15.24%), Sphingomonadaceae (9.23%), and Erwiniaceae (4.61%) were most abundant, whereas Streptococcaceae accounted for only 2.29% (Figure 2B). At the genus level, dominant taxa in *C. kiangsu* included *Lactococcus* (35.55%), *Achromobacter* (28.53%), *Weissella* (15.46%), *Cronobacter* (9.50%), and *Klebsiella* (4.77%). In *C. nigricornis* , the dominant genera were *Klebsiella* (20.10%), *Achromobacter* (14.77%), *Sphingomonas* (9.23%), *Enterobacter* (5.77%), and *Pantoea* (4.61%), with lower levels of *Cronobacter* (2.94%) and *Lactococcus* (2.29%). These results show both overlap and strong differences: the two species share several major genera (e.g., *Klebsiella*, *Achromobacter*, *Lactococcus*, *Cronobacter*), but their rankings and relative abundances differ sharply. In addition, *C. kiangsu* is enriched in *Weissella* (a lactic acid bacterium), whereas *C. nigricornis* contains notable levels of *Sphingomonas* and *Pantoea*, which are rare or absent among dominant taxa in *C. kiangsu* (Figure 2C).

**FIGURE 3:**
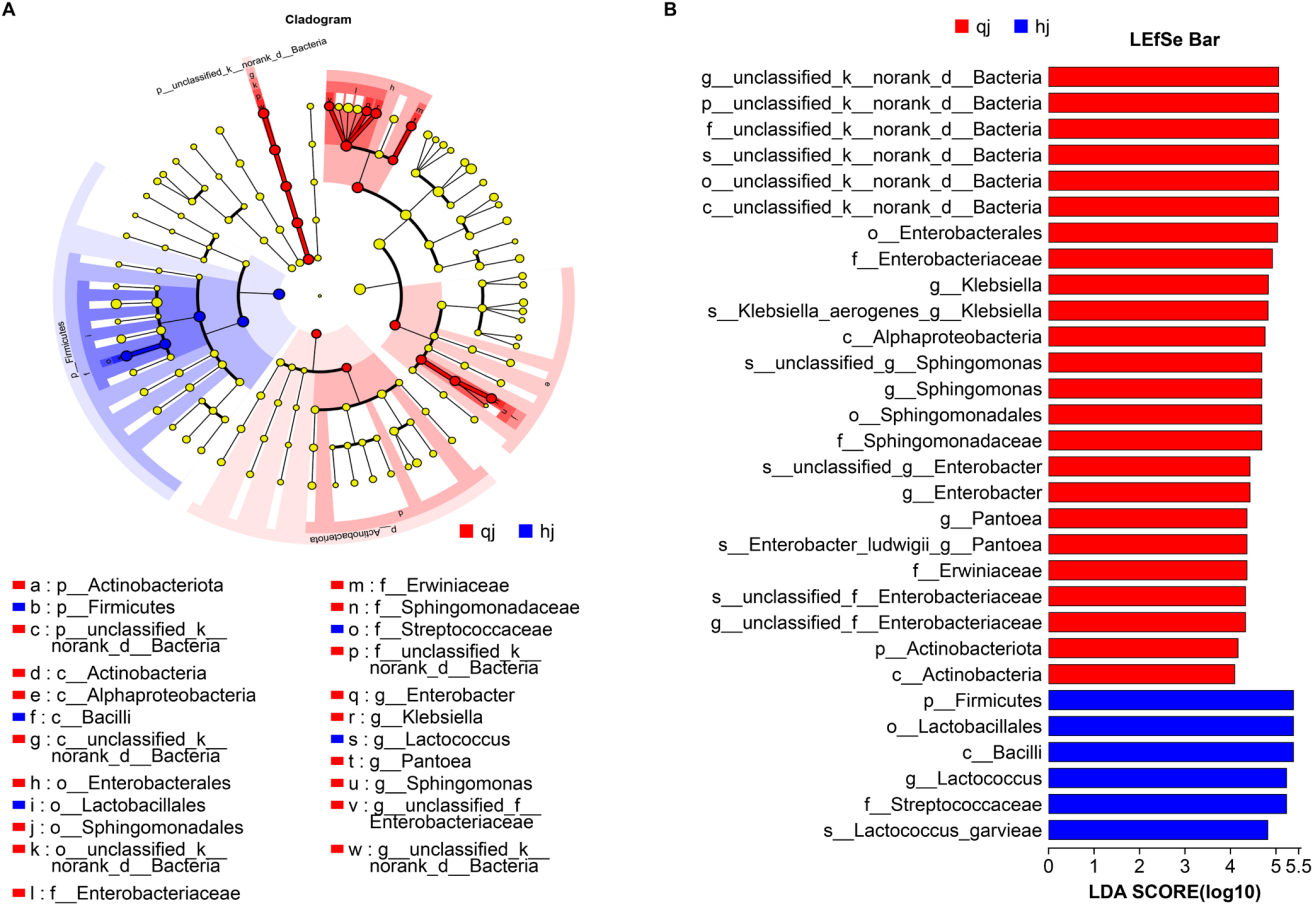
(A) Linear discriminant analysis (LDA) scores showing differences in the relative abundances of gut microbial taxa between *C. kiangsu* (hj) and *C. nigricornis* (qj). Letters p, c, o, f, and u denote phylum, class, order, family, and genus, respectively. (B) Linear discriminant analysis (LDA) identifying distinct bacterial biomarkers in the gut communities of *C. kiangsu* (hj) and *C. nigricornis* (qj).

LEfSe analysis supported these patterns, identifying multiple taxa with significant differences across taxonomic ranks (LDA > 4, *p* < 0.05). Compared with *C. kiangsu*, *C. nigricornis* showed a larger number of enriched discriminatory taxa, particularly within Actinobacteriota and its subordinate lineages (Figure 3A,B). This is consistent with the higher OTU richness observed in *C. nigricornis* and suggests that it harbors a broader set of lower‐abundance but species‐informative taxa.

The observed taxonomic differences indicate distinct gut microbial community configurations between the two host species. Closely related herbivores that share resources often experience strong competition, and higher ecological performance can determine which species dominates a niche (Zhang et al. 2022). Although *C. kiangsu* showed lower overall diversity than *C. nigricornis* , its gut community was strongly dominated by taxa that are frequently linked to digestion and host support, including lactic acid bacteria (e.g., Streptococcaceae/Leuconostocaceae) and Enterobacteriaceae. For example, *Klebsiella* has been reported in multiple insect hosts and may contribute to nitrogen acquisition, plant fiber degradation, and the production of vitamins or amino acids, thereby supporting host growth and reproduction (Yang et al. 2023; Korsa et al. 2022; Cai et al. 2020; Zhang et al. 2023; Rashid et al. 2018). Similarly, *Achromobacter* and lactic acid bacteria such as *Lactococcus* and *Weissella* have been associated with improved digestion and protection against pathogens in other insect systems. The dominance of these taxa in *C. kiangsu* identifies candidate bacterial groups that may be relevant to host digestion and physiology, although their functions were not directly tested in the present study. In contrast, *C. nigricornis* harbored a richer community containing a larger number of low‐abundance taxa, but the ecological significance of this pattern remains unclear. The potential functional implications of these taxonomic differences were further explored using PICRUSt2 predictions, as described below.

### 3.3 Functional Prediction of the Gut Microbiota

**FIGURE 4:**
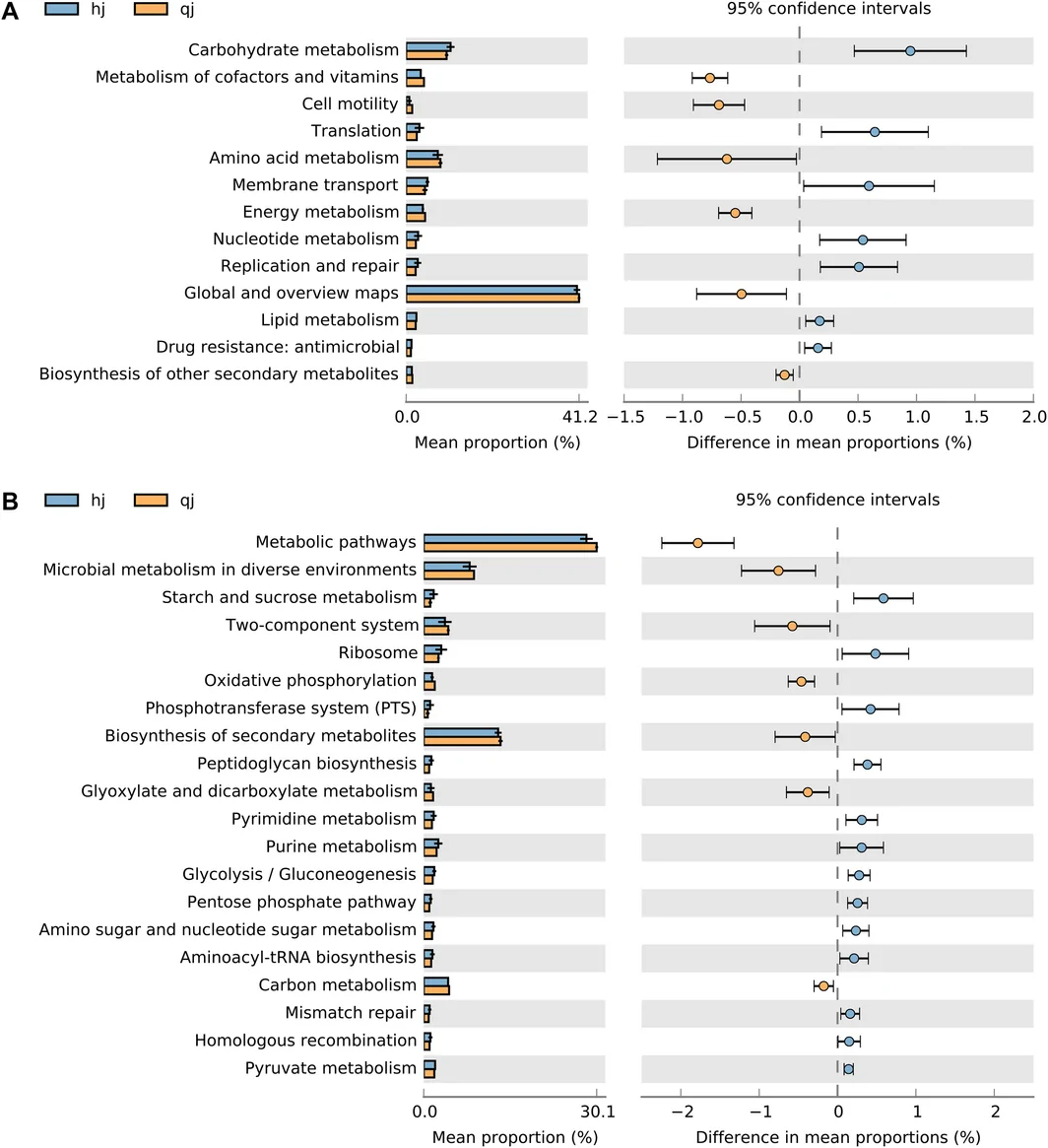
KEGG functional predictions based on PICRUSt2: (A) Level 2 and (B) Level 3. Only gene functions with a relative abundance > 1% and showing significant differences between the gut microbiota of *C. kiangsu* (hj) and the *C. nigricornis* (qj) are displayed.

To explore potential functional consequences of microbiome differences, we performed PICRUSt2‐based functional prediction using KEGG annotations (Figures S2–S4). Across both species, predicted functions were dominated by metabolism‐related pathways. At KEGG Level 1, metabolism accounted for ~75.8% of total predicted functions, far exceeding other categories such as environmental information processing (~7.5%), genetic information processing (~6.0%), and cellular processes (~5.3%). At KEGG Level 2, 45 functional categories were annotated, and the most abundant were global/overview maps (~38.6%), carbohydrate metabolism (~9.3%), amino acid metabolism (~7.7%), membrane transport (~4.4%), and energy metabolism (~4.0%). At KEGG Level 3, more than 300 pathways/modules were predicted; among these, 26 pathways with > 1% relative abundance were highlighted for statistical comparison (Figure 4A,B).

Several predicted functional categories differed significantly between species. At KEGG Level 2, *C. kiangsu* showed higher relative abundance of seven functions, including carbohydrate metabolism, lipid metabolism, membrane transport, nucleotide metabolism, translation, replication and repair, and antimicrobial drug resistance (rank‐sum test, *p* < 0.05). In contrast, *C. nigricornis* showed higher abundance of six Level 2 functions, including cofactor and vitamin metabolism, cell motility, amino acid metabolism, energy metabolism, global/overview maps, and biosynthesis of other secondary metabolites.

At KEGG Level 3, *C. kiangsu* exhibited significantly higher predicted abundance of 13 pathways, largely related to carbohydrate utilization and core biosynthetic processes. These included starch and sucrose metabolism, glycolysis/gluconeogenesis, the pentose phosphate pathway, pyruvate metabolism, amino sugar and nucleotide sugar metabolism, purine and pyrimidine metabolism, peptidoglycan biosynthesis, the phosphotransferase system (PTS), ribosome, aminoacyl‐tRNA biosynthesis, mismatch repair, and homologous recombination. These pathways are consistent with enhanced potential for nutrient breakdown, energy generation, and macromolecule synthesis. By contrast, *C. nigricornis* showed higher abundance of seven pathways, including broad metabolic pathways, microbial metabolism in diverse environments, two‐component systems, oxidative phosphorylation, biosynthesis of secondary metabolites, glyoxylate and dicarboxylate metabolism, and carbon metabolism. These functions are more closely associated with general metabolic versatility and environmental response. Notably, antibiotic resistance‐related functions at KEGG Level 2 were higher in *C. kiangsu*, which may indicate greater robustness of its gut community under environmental perturbation.

Together, the predicted functions suggest different microbiome “strategies” in the two hosts. The microbiome of *C. kiangsu* appears more strongly oriented toward basic energy and nutrient metabolism, particularly the processing of carbohydrates and lipids, whereas the microbiome of *C. nigricornis* shows relatively stronger signals in broad metabolic and environmental‐response pathways. These predicted pathways are relevant to nutrient and energy metabolism. Insect flight is energetically demanding and may rely on carbohydrates, lipids, or a combination of both as major energy substrates (Beenakkers 1969). *C. kiangsu* is known for its relatively strong migratory performance, with adults reported to fly up to 7.8 km per day (Song et al. 2023). In this context, the higher predicted representation of carbohydrate‐ and lipid‐related pathways in the gut microbiota of *C. kiangsu* is noteworthy. However, PICRUSt2 infers functional potential from 16S rRNA gene‐based taxonomic profiles and does not directly measure microbial gene expression, enzyme activity, metabolite production, or host energy utilization (Douglas et al. 2020). Moreover, microbial taxonomic composition and functional structure may not necessarily vary in parallel because different microbial taxa can possess overlapping functional potentials (Louca et al. 2016). Therefore, these findings should be considered hypothesis‐generating rather than direct evidence that the gut microbiota enhances flight capacity or ecological competitiveness in *C. kiangsu*.

In addition, the higher predicted abundance of antibiotic resistance‐related functions in *C. kiangsu* may reflect differences in the genomic composition of its gut microbial community. However, whether these predicted functions correspond to greater microbial community stability or resistance to environmental perturbation remains to be tested experimentally.

**FIGURE 5:**
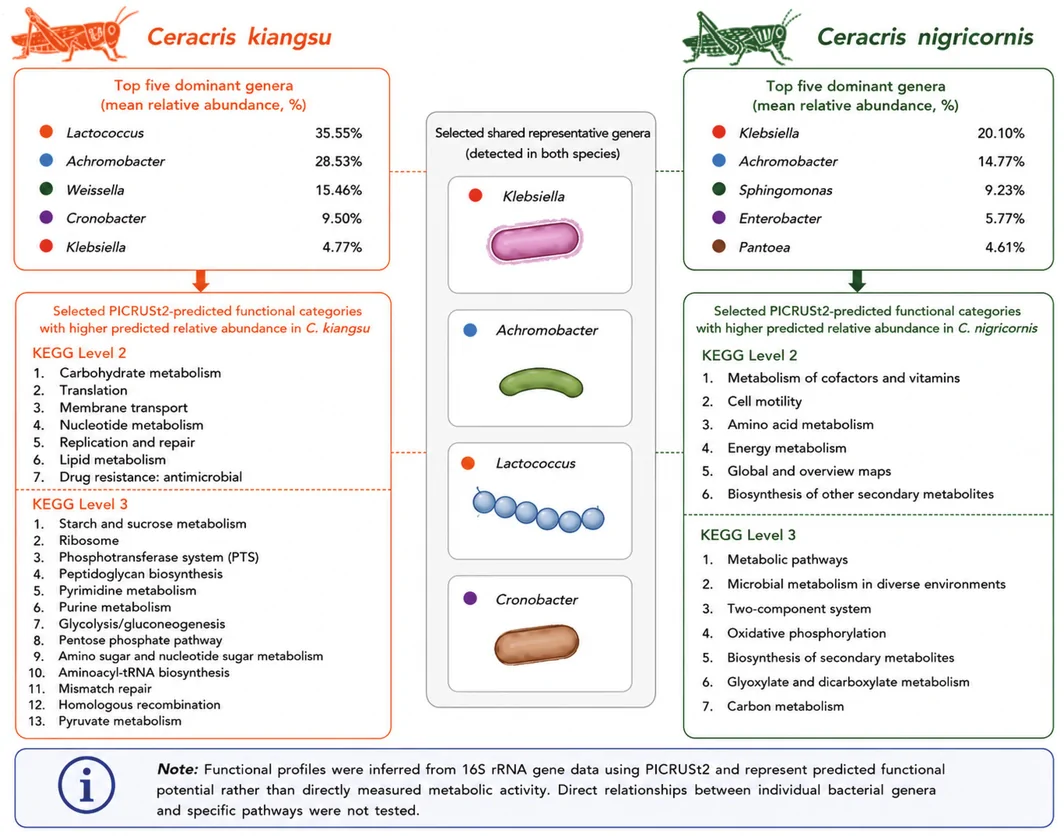
Integrated comparison of representative dominant bacterial genera and selected PICRUSt2‐predicted functional categories in the gut microbiota of *Ceracris kiangsu* and *Ceracris nigricornis*. The left panel shows the representative dominant genera and functional categories with relatively higher predicted abundance in *C. kiangsu*, whereas the right panel shows those of *C. nigricornis* . The middle panel lists representative genera detected in both species. Percentages indicate the mean relative abundances of bacterial genera calculated across all samples of each species. Functional profiles were inferred from 16S rRNA gene data using PICRUSt2 and represent predicted functional potential rather than directly measured metabolic activity. The bacterial icons are schematic representations of typical cellular morphologies and are not drawn to scale. Direct relationships between individual bacterial genera and specific metabolic pathways were not tested.

Taken together, Figure 5 provides an integrated visual summary of the representative dominant genera and selected PICRUSt2—predicted functional categories in the two bamboo locust species. Although *C. kiangsu* and *C. nigricornis* shared several representative genera, including *Klebsiella*, *Achromobacter*, *Lactococcus*, and *Cronobacter*, their relative abundances differed markedly between the two species. *C. kiangsu* was characterized by higher relative abundances of *Lactococcus*, *Achromobacter*, *Weissella*, and *Cronobacter*, whereas *C. nigricornis* showed higher relative abundances of *Klebsiella*, *Sphingomonas*, *Enterobacter*, and *Pantoea*. Consistent with these compositional differences, the predicted functional profile of *C. kiangsu* was more strongly represented by carbohydrate‐ and lipid‐related functions, whereas that of *C. nigricornis* showed greater representation of amino acid metabolism, energy metabolism, and microbial environmental‐response functions. These patterns summarize comparative differences in microbial community composition and predicted functional potential between the two datasets.

Although the raw sequencing reads from both species were reprocessed using the same bioinformatic workflow, technical variation introduced before sequencing could not be completely eliminated because the two datasets were generated independently (Sinha et al. 2017; McLaren et al. 2019). In addition, the study included only adult females, and the sampling designs were not fully balanced: *C. kiangsu* was represented by pooled samples from multiple geographic populations, whereas the publicly available *C. nigricornis* dataset originated from a single population. Differences in sample processing, diet, environment, geographic origin, and sampling design may therefore have contributed to the observed differences in microbial diversity and taxonomic composition. More generally, host‐associated microbial communities may be shaped by multiple community‐assembly processes, including dispersal, diversification, environmental selection, and ecological drift (Costello et al. 2012; Nemergut et al. 2013). We also acknowledge that 97% OTU clustering provides lower fine‐scale sequence resolution than current ASV‐based approaches; therefore, taxon‐level patterns should be interpreted cautiously, with emphasis placed on community‐level comparisons. Overall, these findings should be regarded as comparative patterns rather than effects attributable exclusively to host species identity.

## 4 Conclusion

In summary, our analyses reveal pronounced differences in both gut microbial composition and predicted function between *C. kiangsu* and *C. nigricornis* . These findings indicate distinct comparative microbial patterns between the two datasets, although technical and sampling differences cannot be completely excluded. Although *C. kiangsu* exhibited lower overall microbial diversity, its gut microbiota showed higher predicted representation of several carbohydrate‐ and lipid‐related pathways. These differences generate testable hypotheses regarding potential microbial associations with host energy metabolism. However, because these functions were inferred from 16S rRNA gene profiles, direct links between gut microbial activity, flight performance, and ecological fitness remain to be experimentally validated. Collectively, the present results provide a comparative framework for future studies investigating potential microbiome contributions to ecological differences between the two bamboo locust species.

## Author Contributions

**Hong‐Yu Liao:** conceptualization (equal), data curation (equal), validation (equal), writing – original draft (equal), writing – review and editing (equal). **Ye‐Song Ren:** writing – review and editing (equal). **Xi Cheng:** data curation (equal). **Hai‐Tao Huang:** investigation (equal). **Meng‐Zhi Jin:** visualization (equal). **Yang Zeng:** writing – original draft (equal), writing – review and editing (equal). **Dao‐Hong Zhu:** writing – original draft (equal), writing – review and editing (equal).

## Funding

This work was supported by Hunan Province Forestry Pest Control Special Fund, Graduate Research and Innovation Program of Hunan Province (CX20251234), Graduate Research and Innovation Project of Central South University of Forestry and Technology (2025CX01090).

## Conflicts of Interest

The authors declare no conflicts of interest.

## Supporting information


**Figure S1:** Relative abundances of gut microbial communities in the *Ceracris kiangsu* (hj) and the *Ceracris nigricornis* (qj) at the phylum (A), class (B).
**Figure S2:** KEGG Level 1 functional predictions (PICRUSt2‐based) for the gut microbiota of *Ceracris kiangsu* (hj) and *Ceracris nigricornis* (qj).
**Figure S3:** KEGG Level 2 functional predictions (PICRUSt2‐based) for the gut microbiota of *Ceracris kiangsu* (hj) and *Ceracris nigricornis* (qj).
**Figure S4:** KEGG Level 3 functional predictions (PICRUSt2‐based) for the gut microbiota of *Ceracris kiangsu* (hj) and *Ceracris nigricornis* (qj).
**Table S1:** OTUs and differential bacterial taxa between *Ceracris kiangsu* (hj) and *Ceracris nigricornis* (qj).
**Table S2:** Alpha diversity metrics and Student's t‐test comparisons between *Ceracris kiangsu* (hj) and *Ceracris nigricornis* (qj).

## Data Availability

The data that support the findings of this study have been deposited into CNGB Sequence Archive (CNSA) (Guo et al. 2020) of China National GeneBank DataBase (CNGBdb) (Chen et al. 2020) with accession number CNP0008191.
