# Supplementary material for: Gut Microbiomes of Two Bamboo Locusts Reveal Divergent Metabolic Potentials
Source: Ecol Evol. 2026 Aug 28;16(9):e74143. doi: 10.1002/ece3.74143 (PMC13522933; doi:10.1002/ece3.74143)
Supplement: Supplementary file 1 [file ECE3-16-e74143-s001.docx]

Supplementary Material

# 1 Supplementary Figures and Tables

## 1.1 Supplementary Figures

**Supplementary Figure 1.** Relative abundances of gut microbial communities in the *Ceracris kiangsu* (hj) and the *Ceracris nigricornis* (qj) at the phylum (A), class (B).

**Supplementary Figure 2.** KEGG Level 1 functional predictions (PICRUSt2-based) for the gut microbiota of *Ceracris kiangsu* (hj) and *Ceracris nigricornis* (qj).

**Supplementary Figure 3.** KEGG Level 2 functional predictions (PICRUSt2-based) for the gut microbiota of *Ceracris kiangsu* (hj) and *Ceracris nigricornis* (qj).

**Supplementary Figure 4.** KEGG Level 3 functional predictions (PICRUSt2-based) for the gut microbiota of *Ceracris kiangsu* (hj) and *Ceracris nigricornis* (qj).

## 1.2 Supplementary Table

| Group | OTUs | Phylum | Class | Order | Order | Genus |
| --- | --- | --- | --- | --- | --- | --- |
| hj | 362 | 25 | 51 | 104 | 163 | 257 |
| qj | 1290 | 20 | 47 | 113 | 177 | 268 |

**Supplementary Table 1.** OTUs and differential bacterial taxa between *Ceracris kiangsu* (hj) and *Ceracris nigricornis* (qj).

| Estimators | hj-Mean | hj-Sd | qj-Mean | qj-Sd | P_value(hj-qj) | P_adjust(hj-qj) |
| --- | --- | --- | --- | --- | --- | --- |
| sobs | 13.933 | 9.0589 | 78 | 15.919 | 4.73E-17 | 2.84E-16 |
| shannon | 0.89946 | 0.56224 | 2.8359 | 0.52617 | 1.87E-10 | 3.75E-10 |
| simpson | 0.57417 | 0.25591 | 0.12436 | 0.070047 | 2.18E-05 | 3.27E-05 |
| ace | 26.699 | 36.118 | 83.974 | 11.651 | 9.65E-05 | 9.65E-05 |
| chao | 17.264 | 13.104 | 82.876 | 12.948 | 8.96E-15 | 2.69E-14 |
| coverage | 0.99893 | 0.00099071 | 0.997 | 0.0013407 | 6.00E-05 | 7.19E-05 |

**Supplementary Table 2.** Alpha diversity metrics and Student’s t-test comparisons between *Ceracris kiangsu* (hj) and *Ceracris nigricornis* (qj).
